# A new metabolic signature contributes to disease progression and predicts worse survival in melanoma

**DOI:** 10.1080/21655979.2020.1822714

**Published:** 2020-10-21

**Authors:** Mengdi Wan, Binyu Zhuang, Xiao Dai, Liang Zhang, Fangqing Zhao, Yan You

**Affiliations:** Department of Dermatology, The Forth Hospital of Harbin Medical University, Harbin, China

**Keywords:** Metabolism, progression, prognosis, melanoma, immune

## Abstract

Metabolic reprogramming is a common hallmark of tumor cells and is a crucial mediator of resistance toward anticancer therapies. The pattern of a metabolism-related signature in melanoma remains unknown. Here, we explored the role of a multi-metabolism-related gene signature in melanoma.We used the training and validation sets to develop a multi-metabolism-related gene signature. Cox regression analysis and the least absolute shrinkage and selection operator (LASSO) method were used for constructing a model. The predictive role of the metabolic signature with clinicopathological features of melanoma was also analyzed. Functional analysis of this metabolic signature was also investigated.A ten metabolism-related gene signature was identified and can stratify melanoma into high- and low- risk groups. The signature was correlated with progressive T stage, Breslow thickness, Clark level, and worse survival (all *Ps*< 0.01). This metabolic signature was shown as an independent prognostic factor and was also a predictive indicator for worse survival in various clinical and molecular features of melanoma. Furthermore, the metabolic signature was implicated in immune responses such as the regulation of T cell activation and cytokine activity. The metabolic signaturewas associated with the progression and worse survival of melanoma. Our study offered a valuable metabolism-targeted therapy approach for melanoma.

## Introduction

Melanoma is a highly malignant and invasive tumor of the skin, it is correlated with significant morbidity and mortality. In 2018, global cancer statistics report that an estimated 287,723 new cases will be diagnosed, and approximately 60,712 people will die due to melanoma in the world [1].Although the treatment strategies have some improvements, the recurrence rate of melanoma is still high and most patients achieve short term survival [2,3]. Therefore, the understanding and identification of novel biomarkers to control melanoma progression and prognosis are of importance to provide new therapeutic insights.

Many studies have reported the molecular mechanisms are closely linked to melanoma [4,5]. To gain and maintain the capacity of cell proliferation, tumor cells need to activate or enhance metabolic pathways in order to fulfill their energy requirements. Abnormality of metabolism has been recognized as a defining hallmark of cancer [6,7]. Genetic alterations in oncogenes and tumor suppressors involve in metabolic reprogramming in cancer. Dysregulated metabolism contributes to tumor cell survival and growth, thereby resulting in malignant transformation and cancer progression [8,9]. Reprogrammed metabolism also promotes tumor cells to survive and proliferate in harsh microenvironmental conditions by regulating tumor angiogenesis and sabotaging cancer immunity [10–12]. Targeting metabolism may represent an attractive target for therapeutic strategies for cancer therapy [13]. Recently, several studies have begun to investigate the prognostic significance of the metabolism-related signature for predicting prognosis in several tumors such asglioma and glioblastoma [14,15]. However, role of the metabolism-related signature in melanoma is still unclear.

In this study, we first identified the metabolism-related gene signature in melanoma. Then, we evaluate and validate whether the metabolism-related gene signature was a reliable molecular feature for predicting survival in multiple datasets of melanoma samples. This study might provide a new insight for understanding the metabolic mechanism of melanoma and support the development of a novel drug target strategy to treat melanoma.

**Figure:**
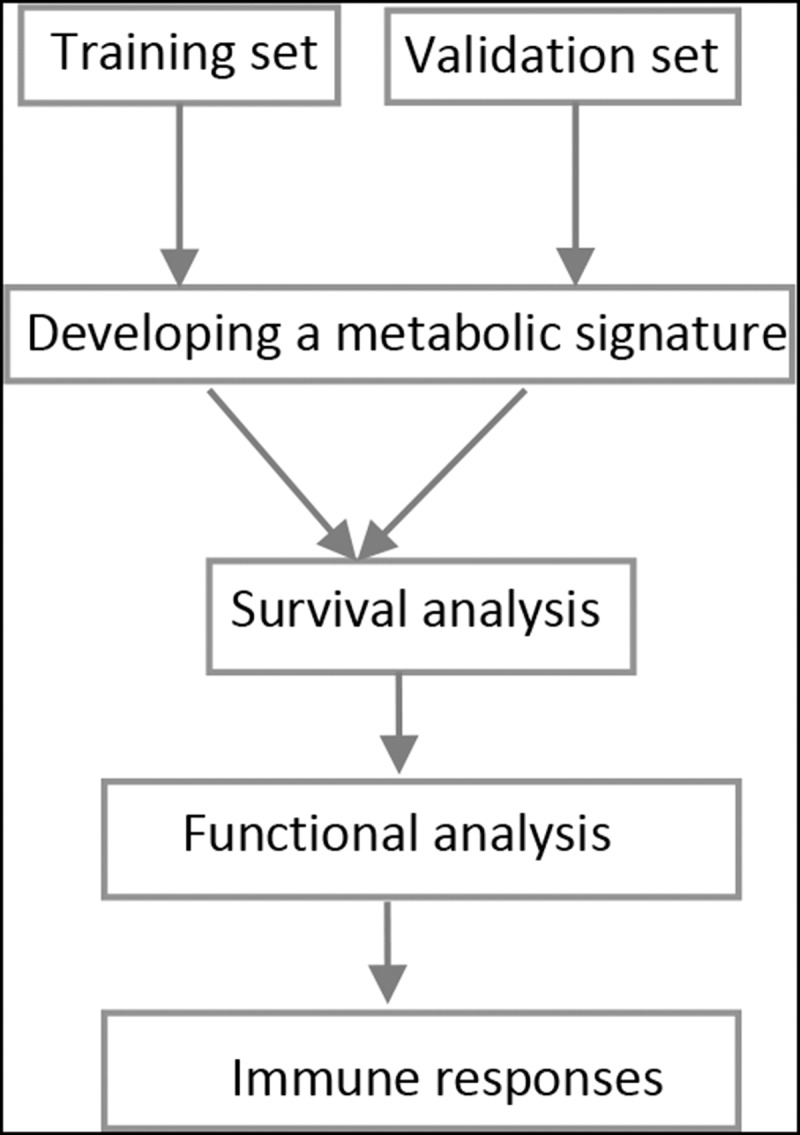

## Materials and methods

### Sample information

The raw mRNA expression profiling data (Workflow Type: HTSeq-Counts) and corresponding clinical information for cases with melanoma were obtained from The Cancer Genome Atlas (TCGA) following the approval of this project. The methods of biospecimen collection and RNA isolation were previously described by this project [16]. Expression counts were normalized by using the Trimmed Mean of M-values (TMM) method [17] and the mean levels of gene expression with ≤1 in total samples were excluded.The normalized expression values were transformed via a log2(x +1).Patients with melanoma that did not have available survival data were excluded from the analysis. The clinicopathological characteristics included age at initial pathologic diagnosis, gender, clinical stage, T stage, lymph node metastasis, distant metastasis, Clark level, and Breslow thickness. Finally, 458 melanoma samples with available survival information were identified.

Microarray expression datasets were obtained from the Gene Expression Omnibus (GEO). GSE19234 and GSE53118 datasets with available survival information and sample sizes with > 40 were collected. Patients without available survival data were excluded. Normalized GSE19234 data were conducted from the GPL570 platform [HG-U133_Plus_2] Affymetrix Human Genome U133 Plus 2.0 Array. Normalized GSE53118 datawere conducted from the GPL6884 platform Illumina HumanWG-6 v3.0 expression beadchip. If a gene symbol mapped to multiple probes, the average value was applied as its expression level. For GSE19234 dataset, we also performed a log2 transformation. To get a larger study population, the ComBat method was applied to remove the batch effects and combine these two datasets [18]. Eventually, 123 melanoma samples with available survival information were identified.

TCGA data with 458 melanoma samples were used as a training cohort. Moreover, the GEO microarray expression dataset (n = 123 melanoma samples) was used as a validation set. Overall survival (OS) was recorded as the time from study enrollment to the date of death owing to any cause or the last follow-up time. The baseline characteristics are showed in Table 1.

**Table 1.** Baseline characteristics of the study population

Training set				Validation set	
Characteristics		Number of cases	%	Number of cases	%
Age	Median: 58 (15–90 years)			Median: 58 (18–92 years)	
	≥ 60	219	47.8	58	47.2
	< 60	239	52.2	65	52.8
Gender					
	Male	284	62	78	63.4
	Female	174	38	45	36.6
Tumor stage					
	Stage 3–4	191	45.3	123	100
	Stage 0–2	231	54.7	0	0
T classification					
	T 3–4	240	62		
	T 0–2	147	38		
Distal metastasis					
	Positive	23	5.3		
	Negative	409	94.7		
Lymph node metastasis					
	Positive	176	43.6		
	Negative	228	56.4		
Clark level					
	IV–V	217	68.9		
	I–III	98	31.1		
Breslow thickness					
	> 1.5 mm	249	70.5		
	≤ 1.5 mm	104	29.5		
Ulceration					
	Positive	165	53.4		
	Negative	144	46.6		

### Metabolism-related genes finding

The Hallmark gene sets, which represent specific well-defined biological states or processes, were collected from the Molecular Signatures Database (MSigDB) (https://www.gsea-msigdb.org/gsea/msigdb/index.jsp). The metabolism-related genes were obtained from the Hallmark gene sets, including BILE_ACID_METABOLISM, FATTY_ACID_METABOLISM, GLYCOLYSIS, HEME_METABOLISM, and XENOBIOTIC_ METABOLISM.

### Development of the metabolism-related gene signature for melanoma

The following steps were applied to develop the signature. First, in the training and validation cohorts, the metabolism-related geneswere performed using univariate Cox regression analysis (Table S1). The univariate results with *P *< 0.05 were selected to obtain overlapping prognostic genes and 30 significant prognostic genes were found (Table S2). Second, in order to get the final metabolism-related prognostic genes in the present model, these 30 prognostic genes were then conducted using the least absolute shrinkage and selection operator (LASSO) method in the training cohort, showing 16 selected genes (Figure S1). Finally, multivariate Cox regression analysis (two-step method: both forward and backward steps) [19]was performed for these 16 candidate genes. Then, the final 10 genes were identified for constructing our signature.

### The functional analysis of this metabolic signature

The relationship between genes and the multi-metabolism gene signature was analyzed, the results of the Spearman’s correlation coefficient with the absolute values of > 0.5 were selected for significant genes. Then, functional annotation analysis of this multi-metabolism gene signature was performed using ‘clusterProfiler’ package [20].

### Statistical analysis

Data analysis was conducted with the R software version 3.5.1 (R Foundation for Statistical Computing, Vienna, Austria). Differences betweenthe multi-metabolism gene signature and clinical and pathological groups such as age (≥ 60 vs. < 60 years), gender (male vs. female), tumor stage (stage 3 −4 vs. stage 0 −2), lymph node metastasis (positive vs. negative), distant metastasis (positive vs. negative), T classification (T 3 −4 vs. T 0 −2), Breslow thickness (>1.5 mm vs. ≤1.5 mm), Clark level (IV−V vs. I−III)were calculated using the Wilcoxon signed-rank test. The time-dependent receiver-operating characteristic (ROC) curve and area under the curve (AUC) [21]were applied to evaluate the predictive accuracy of the multi-metabolism gene signature. The cutoff values of the multi-metabolism gene signature were determined based on the median value of the training set (cutoff = 1.0016).Subsequently, cases were divided into high- and low-risk groups according to the cutoff value of risk score of this signature.Kaplan-Meier survival analysis and log-rank test were performed to compare differences for the low- and high-risk groups. UnivariateCox proportional-hazards regression analysiswas conducted to analyze the association of the multi-metabolism gene signature with the survival (hazard ratio: HR and 95% confidence interval: 95% CI). The results from univariate survival analysis were selected as the candidate variables when *P* values were <0.1. Multivariate Cox proportional-hazards regression analysis was further carried out using the candidate prognostic parameters such asage, clinical stage, T stage, lymph node metastasis, Breslow thickness, and Clark level.

## Results

### Study characteristics

As shown in Table 1, a total of 458 melanomas (284 males and 174 females) were enrolled in the TCGA training set. The median age at initial pathologic diagnosis was 58 years old. The median follow-up time for survival analysis at last contact was 34.9 months, with a range from 0 to 369.9 months in the training set.An independent validation set (n =123 melanoma samples) was further used. The median agewas 58 years old and the median follow-up time was 57.79 months (range: 2.2 to 402.86 months) for the validation set.

### Construction of a metabolism-related gene signature

After using univariate Cox regression analysis, LASSO method, and multivariate Cox regression analysis, ten metabolism-related genes were finally identified for constructing our signature in the training set. A prognostic index was established based on the expression value of each metabolism-related gene and its corresponding regression coefficient (Table S3).

### A ten metabolism-related gene signature in melanomaprogression

**Figure 1.**
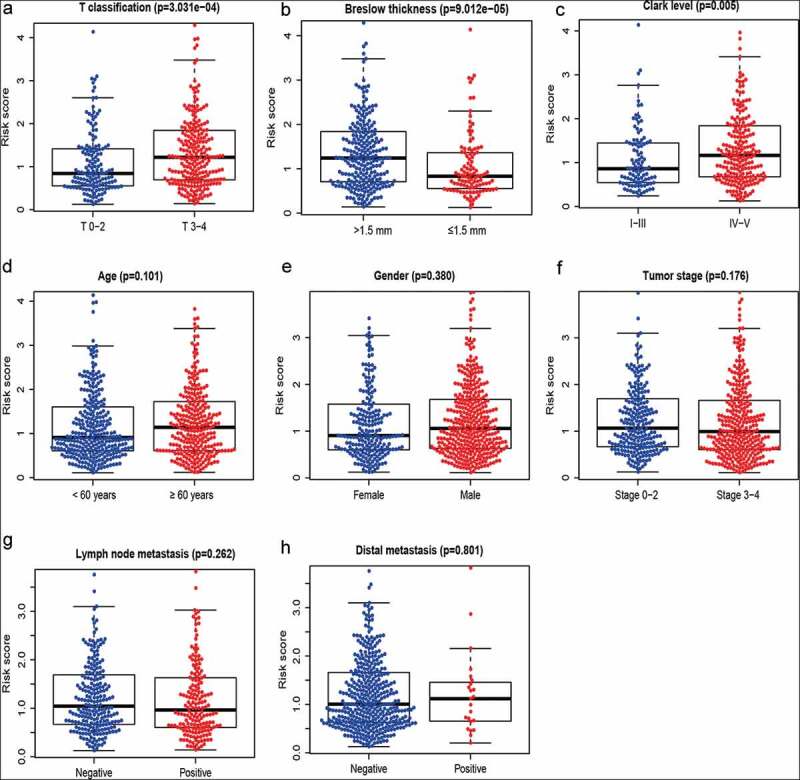
A metabolism-related gene signature in melanoma. (a) T classification; (b) Breslow thickness; (c) Clark level; (d) Age; (e) Gender; (f) Tumor stage; (g) Lymph node metastasis; (h) Distant metastasis

We investigated whether the metabolism-related gene signature was associated with pathologic characteristics in the whole set. The ten metabolism-related gene signaturewas significantly higher in advanced T stage compared with early T stage (*P *< 0.001) (Figure 1(a)).This ten metabolism-related gene signature was also higher in patients with Breslow thickness >1.5 mm compared with Breslow thickness ≤ 1.5 mm (*P *< 0.001) (Figure 1(b)) and significantly higher in Clark level IV–V than in Clark level I–III (*P* = 0.005) (Figure 1(c)), suggesting that our metabolism-related gene signaturemay be associated with melanoma progression. No correlation was found between the metabolism-related gene signature and other clinical features (Figure 1(d–h)).

### A metabolism-related gene signaturefor predicting survival in melanoma

**Figure 2.**
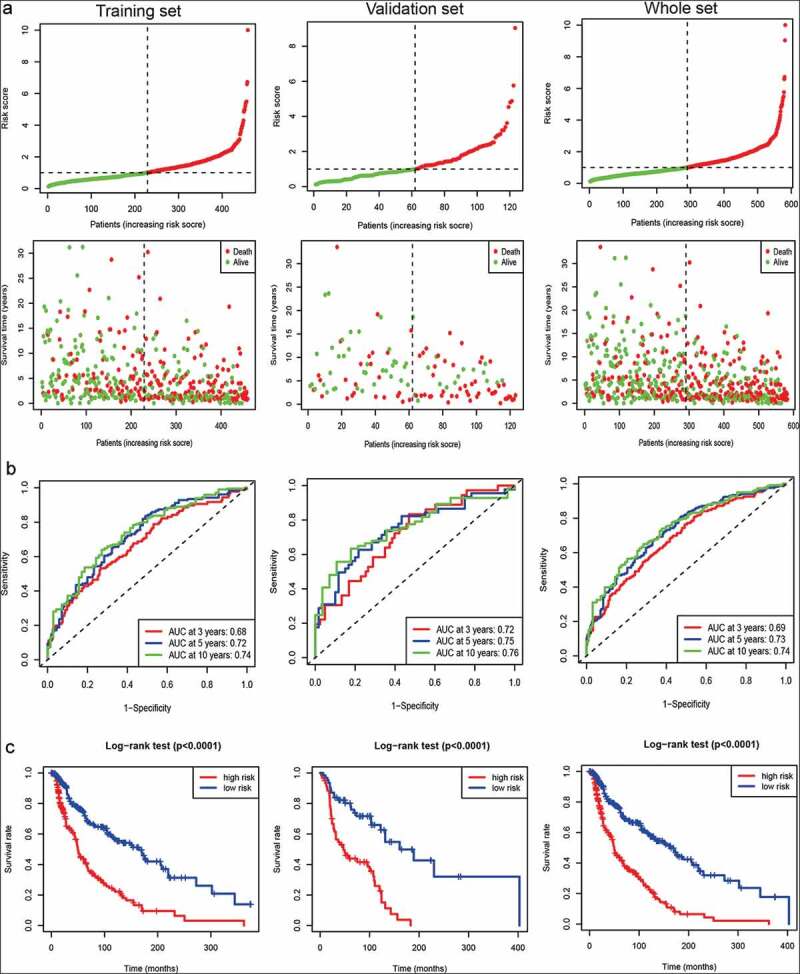
Survival prediction of the metabolism-related gene signature in the training set, the validation set, and the whole set of melanoma samples. (a) Samples sorted by risk score and the corresponding survival status. (b) ROC curve of the metabolism-related gene signature. (c) The Kaplan–Meier curves of metabolism-related gene signature (the high- and low-risk groups)

The distribution of the risk score and survival status of each sample were displayed in the training set, the validation set, and the whole set (Figure 2(a)), suggesting the increasing risk score with shorter survival. Time-dependent ROC analyses were used to evaluate the predictive accuracy at 3, 5, and 10 years. By computing AUC values, the results showed that AUC valuesfor the long-time follow-up at 5 and 10 years were > 0.70 in each set (Figure 2(b)), suggesting the powerful ability of our metabolism-related gene signature for predicting prognosis in melanoma. Additionally, Kaplan-Meier curve demonstrated that melanoma samplesamong thehigh-risk group had a shorter survival than those amongthe low-risk group (all *Ps*< 0.0001) (Figure 2(c)).

### Independent prognostic value of a metabolism-related gene signaturein melanoma

The univariate and multivariate survival analyses were performed to determine whether this ten metabolism-related gene signature may predict poor prognosis of patients with melanoma (Table 2). The univariate Cox analysis showed that the high-risk group was closely associated with worse survival (the training set: HR = 2.72, 95% CI = 2.05–3.62, *P *< 0.0001).After adjusting for age, clinical stage, T stage, lymph node metastasis, Breslow thickness, Clark level, and ulceration,our metabolism-related gene signature remained independently correlated with poor survival, with a HR of 2.00 (95% CI: 1.32–3.02, *P* = 0.001) in the training set.Moreover, ulceration was also an independent prognostic factor(HR = 1.64, *P* = 0.017).

**Table 2.** Univariate and multivariate Cox analyses of the metabolism-related signature

Variables	HR with 95% CI	*P*
Training set		
Univariate analysis		
Our signature (high vs. low)	2.72 (2.05–3.62)	< 0.0001
Age (≥ 60 vs. < 60 years)	1.62 (1.22–2.16)	0.0008
Gender (male vs. female)	1.17 (0.87–1.57)	0.29
Tumor Stage (stage 3–4 vs. stage 0–2)	1.72 (1.28–2.32)	0.0004
T classification (T 3–4 vs. T 0–2)	1.95 (1.42–2.66)	< 0.0001
Distal metastasis (positive vs. negative)	1.64 (0.83–3.20)	0.152
Lymph node metastasis (positive vs. negative)	1.77 (1.31–2.40)	0.0002
Breslow thickness (>1.5 vs. ≤ 1.5 mm)	2.23 (1.58–3.17)	< 0.0001
Clark level (IV–V vs. I–III)	2.11 (1.47–3.03)	< 0.0001
Ulceration (positive vs. negative)	1.98 (1.40–2.81)	0.0001
Multivariate analysis		
Our signature (high vs. low)	2.00 (1.32–3.02)	0.001
Age (≥ 60 vs. < 60 years)	1.07 (0.71–1.62)	0.7444
Tumor Stage (stage 3–4 vs. stage 0–2)	0.86 (0.26–2.81)	0.8058
T classification (T 3–4 vs. T 0–2)	0.93 (0.46–1.88)	0.8466
Lymph node metastasis (positive vs. negative)	2.83 (0.86–9.27)	0.0858
Breslow thickness (>1.5 vs. ≤ 1.5 mm)	1.42 (0.66–3.05)	0.3709
Clark level (IV–V vs. I–III)	1.21 (0.73–1.99)	0.4604
Ulceration (positive vs. negative)	1.64 (1.09–2.45)	0.017
Validation set		
Univariate analysis		
Our signature (high vs. low)	3.59 (2.13–6.06)	< 0.0001
Age (≥ 60 vs. < 60 years)	1.53 (0.95–2.46)	0.0802
Gender (Male vs. female)	0.99 (0.61–1.61)	0.982
Multivariate analysis		
Our signature (high vs. low)	3.44 (2.02–5.86)	< 0.0001
Age (≥ 60 vs. < 60 years)	1.23 (0.76–1.99)	0.404

HR: hazard ratio; CI: confidence interval.

In the independent validation set, the univariate Cox analysis demonstrated that the high-risk group was correlated with poor survival (HR = 3.59, 95% CI = 2.13–6.06, *P *< 0.0001). Further multivariate Cox analysis suggested that our metabolism-related gene signature remained an independent prognostic factor (HR = 3.44, 95% CI = 2.02–5.86, *P *< 0.0001).

### Predictive value of the metabolism-related gene signature with the survival in different pathological variables

**Figure 3.**
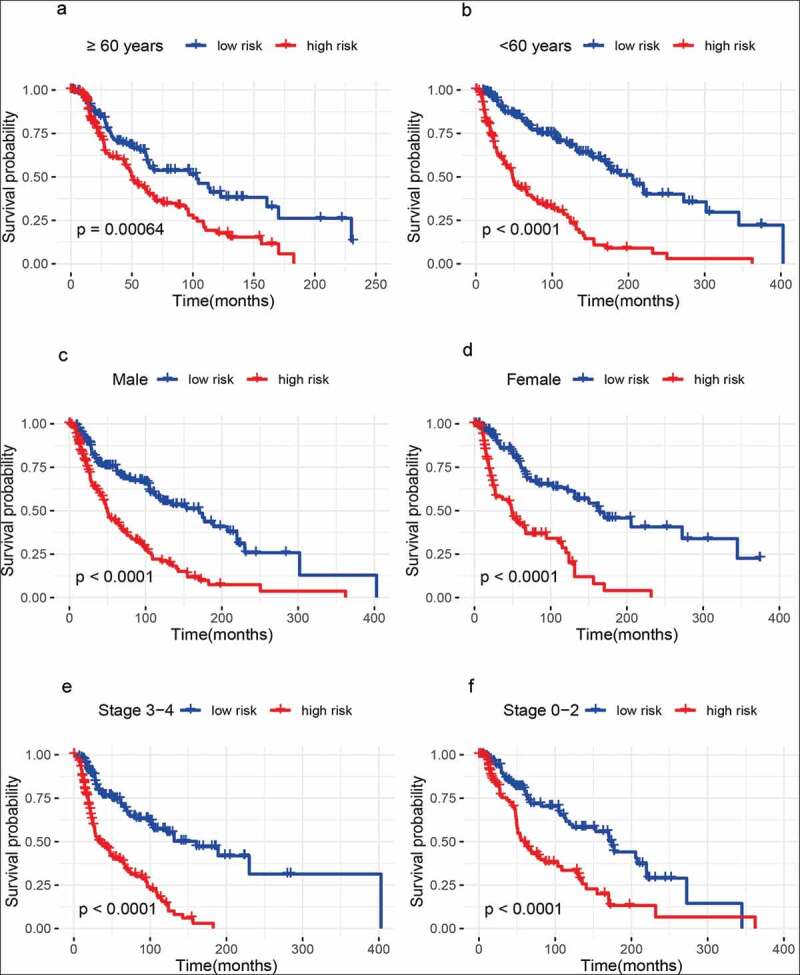
Outcome prediction of the metabolism-related gene signature for melanoma based on age, gender, and tumor stage. (a) ≥ 60 years; (b) < 60 years; (c) Male samples; (d) Female samples; (e) Stage 3 − 4 melanoma; (f) Stage 0 − 2 melanoma

**Figure 4.**
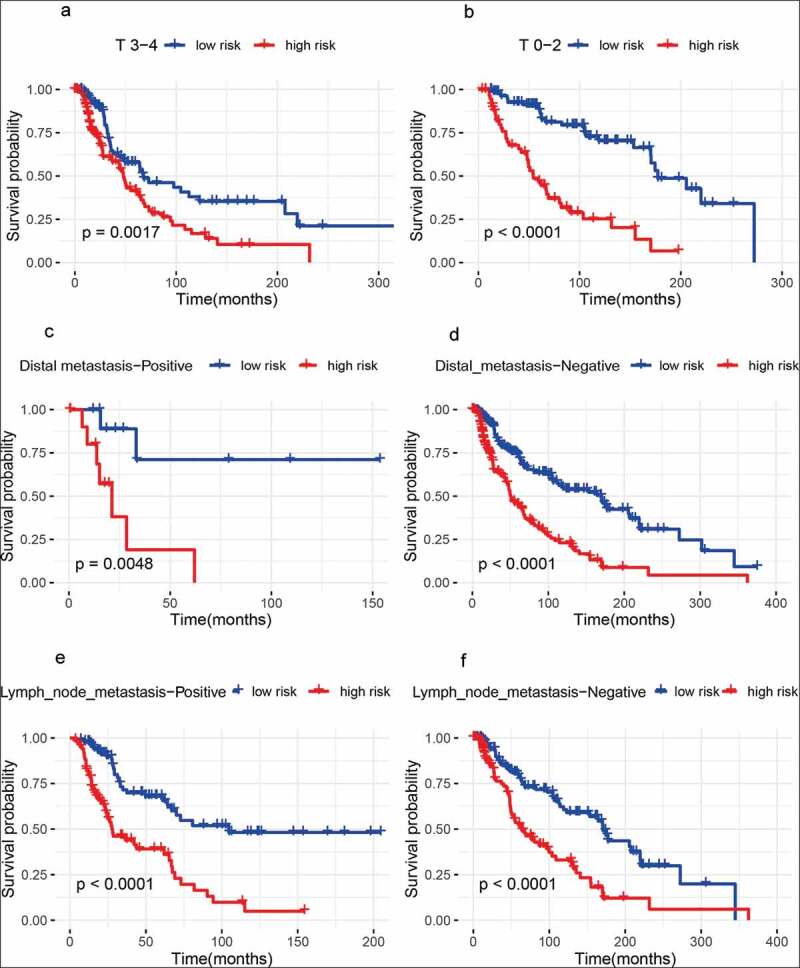
Outcome prediction of the metabolism-related gene signature for melanoma based on T classification, lymph node metastasis, and distant metastasis. (a) T 3 − 4 melanoma; (b) T 0 − 2 melanoma; (c) samples with distant metastasis; (d) samples without distant metastasis; (e) samples with lymph node metastasis; (f) samples without lymph node metastasis

**Figure 5.**
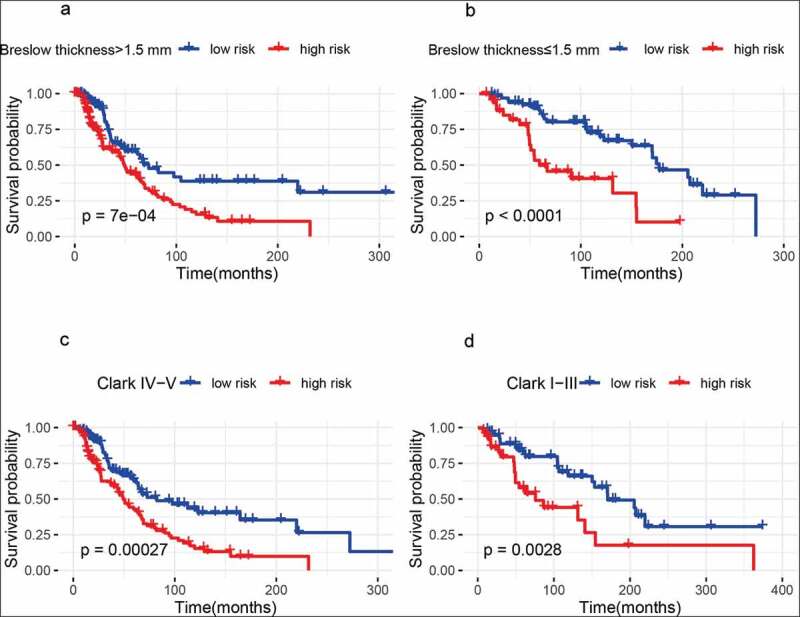
Outcome prediction of the metabolism-related gene signature for melanoma based on Breslow thickness and Clark level. (a) Breslow thickness >1.5 mm; (b) Breslow thickness ≤1.5 mm; (c) Clark IV−V; (d) Clark I− III

We further investigated whether the metabolism-related gene signature was associated with the prognosis in different pathological variables of melanoma patients. Thus, stratification analyses based on age (older: ≥ 60 years and younger: < 60 years), gender (male and female), clinical stage (stage 3–4 and stage 0–2), T classification (T 3–4 and T 0–2), distant metastasis (positive and negative), lymph node metastasis (positive and negative), Breslow thickness (>1.5 mm and ≤ 1.5 mm), Clark level (IV–V and I–III), and ulceration(positive and negative)were carried out in the whole set.Our results showed that 10 metabolism-related gene signature was also correlated with shorter survival among older or younger patients, male or female patients, clinical stage 3–4 or stage 0–2 patients, patients with T 3–4 or T 0–2, patients with lymph node metastasis or without lymph node metastasis, patients with distant metastasis or without distant metastasis, patients with Breslow thickness >1.5 mm or with Breslow thickness ≤ 1.5 mm, patients with Clark level IV–V or with Clark level I–III (all *Ps*< 0.01) (Figures 3–5), and patients with ulceration or without ulceration (Figure S2 A-B),which suggested that this metabolism-related gene signatureremained a powerful tool for survival prediction in each stratumof age, gender, clinical stage, T classification, distant metastasis, lymph node metastasis, Breslow thickness, Clark level, andulceration.

### Identification of the metabolism-related gene signature-related process

**Figure 6.**
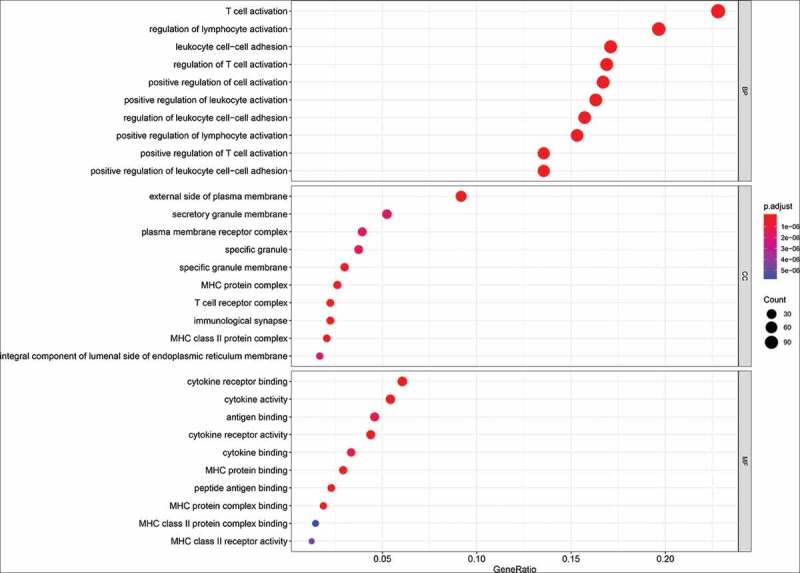
The functional analysis of the metabolism-related gene signature. MF: molecular function; BP: biological process; CC: cellular component

Our analysis showed thatthis metabolism-related gene signature involved in the regulation of T cell activation, regulation of leukocyte cell−cell adhesion, antigen binding, MHC protein complex, cytokine activity, and plasma membrane receptor complexetc. (Figure 6).

## Discussion

Accumulating evidence suggests that metabolism deregulation is one of the emerging hallmarks of tumor [22]. Abnormality of metabolism is correlated with cancer progression, due to its crucial role in cell growth, proliferation, survival, angiogenesis, and invasion.Oncogenes or tumor suppressors in tumor have been shown as regulators of metabolism [8,9,23,24]. Metabolic reprogramming has been demonstrated in cancer stem cells (CSCs), which are responsible for the property of self-renewal and resistant to chemotherapy and radiation. Thus, metabolic reprogramming is a key mediator of resistance toward anticancer therapies. Targeting metabolism could be applied to improve the efficacy of cancer therapy [13,25,26]. Metabolomic alterations have been reported in melanoma and are shown to be involved in melanomaprogression and metastasis [27,28]. However, the role of the metabolism-related gene signature in melanoma is still largely unknown. To the best of our knowledge, this was the first study to explore the metabolism-related gene signature and its potential survival effect on melanoma. The pattern of the metabolism-related gene signature mediating metabolic reprogramming in melanoma was the focus on the current work.

In this work, to diminish bias, each experiment was performed at least two times to ensure that all data were adequately truthful to the content of all cases included. Moreover, multivariate Cox proportional-hazards regression analysis was conducted after controlling for age, clinical stage, T stage, lymph node metastasis, Breslow thickness, ulceration, and Clark level.We found that our metabolism-related gene signature was correlated with melanoma progression and survival. This metabolism-related gene signature was shown to be an independent prognostic factor for predicting survival in melanoma. Further mechanism suggested that this metabolic signature involved in immune regulation and plasma membrane receptor complex etc.

Recently, the metabolic signature has been reported in cancer [29]. A lipid metabolism gene signature in the high-risk patients is correlated with worse survival and it can be an independent prognostic factor in diffuse glioma [14]. A high glycolysis-based ten-gene signature score predicts poor prognosis of glioblastoma [15]. A metabolic gene signature mediates dedifferentiation and progression of papillary thyroid cancer [23]. In the current study, the similar findings of these previous studies in other tumors were consistent with our results and we found that our metabolism-related gene signature was related to the progression of melanoma and this signature in the high-risk group showed a worse survival and could become a useful prognostic factor in melanoma.Ulceration has been reported as a prognostic factor in melanoma [30]. In our study, we found that this metabolism-related gene signature and ulceration weresignificant independent prognostic factors in melanoma. To further evaluate the prediction ability of our signature and ulceration,our signature had a higher AUC value (0.744) compared with ulceration (0.651) (Figure S2 C). Our results suggested that this metabolism-related gene signature may have a promising predictive capacity through comparing with pathological features such as ulceration.Targeting metabolism have been effective strategies for cancer therapies [13,31].This signature couldhave the potential topredict clinical benefit from drugs targeting enzymes or metabolites and/or combination treatments. Additional experimental researches are required to predict response for metabolism-targeted therapiesin the future.

The immune system plays a crucial role in the defense against pathogens and the maintenance of tissue homeostasis [32]. Metabolic pathways influence immune cell function and fate. Metabolism has an important role in the regulation of immune responses [32,33]. A previous study reported that an energy metabolism-related signature was involved in the immune and inflammatory responses in diffuse glioma [34]. We found that our metabolism-related gene signature was mainly related to the regulation of T cell activation and leukocyte cell−cell adhesion, antigen binding, MHC protein complex, cytokine activity, etc., indicating that this metabolic signature involved in immune regulation.

Melanoma is ahighly aggressive and metastatic cancer. Approximately 20% of melanoma patients will develop metastatic disease, with an extremely poor prognosis [35,36]. The development of effective management strategies of metastatic melanoma using specific markers is required to improve clinical intervention and the survival of patients [37]. In this work, we demonstrated that our metabolism-related gene signature was a powerful predictor for worse prognosis in melanoma patients with lymph node metastasis or without lymph node metastasis and with distant metastasis or without distant metastasis, indicating that this signature could serve as a potential molecular tool for metastatic melanoma. Additionally, we also foundthat this signature remained a strongpredictor for survival in other different clinical and pathological characteristics.

This work had several limitations.First, this study was a retrospective design.Second, the missing rate of the clinical variables was high in the validation set, which may result in a decreased statistical power in multivariate Cox proportional-hazards regression analysis.Third,we evaluated whether our signature could potentially predict survival in an early stage T1 melanoma without lymph node metastasis. This signature could not predict survival (Figure S2 D), which may be due to small sample sizes (n =29).Additional studies with large sample sizes are needed to validate this result in the future.

In the future, the effect of this metabolism-related gene signature in melanoma for predicting survival and developing novel drug combination strategies should be further clarified via more experimental researches. Additionally, relevant study from more countries is needed to further validate whether this signature has a superior prediction capacity through comparing with other signatures and clinical characteristics.

## Conclusion

In conclusion, our findings provided the first evidence to support that a metabolism-related gene signature was related to the progression and worse survival of melanoma. This new signature was not only predictive of melanoma but also closelyrelated to poor clinical outcomes of patients with melanoma. Our findings offered a new understanding of metabolic regulation and provided a valuable metabolism-targeted therapy approach in melanoma. Further research is needed to elucidate the role of a metabolic signature in melanoma.

## Supplementary Material

Supplemental Material

## Data Availability

The datasets used and/or analyzed during the current study are available from the corresponding author on reasonable request.

## Highlights

1) This metabolic signature was related to melanoma progression.

2) This metabolic signature involved in immune responses.

3) This metabolic signature could predict survival in melanoma.
