# Supplementary material for: A new metabolic signature contributes to disease progression and predicts worse survival in melanoma
Source: Bioengineered. 2020 Oct 21;11(1):1099–111. doi: 10.1080/21655979.2020.1822714 (PMC8291831; doi:10.1080/21655979.2020.1822714)
Supplement: Supplemental Material [file KBIE_A_1822714_SM0229.zip › Supplemental caption.docx]

**Supplemental materials**

Figure S1. Coefficient profiles and a partial likelihood deviance plot of LASSO method.

Figure S2. Predictive ability of the metabolism-related gene signature and ulceration in melanoma. (A) Survival prediction of this signature in patients with ulceration; (B) Survival prediction of this signature in patients without ulceration; (C) Predictive value of this signature and ulceration; (D) Survival prediction of this signature in an early stage T1 melanoma that is node negative.

Table S1. 675 metabolism-related genes in the training and validation sets.

Table S2. 30 metabolism-related genes with a significant survival.

Table S3. Ten metabolism-related genes for constructing a model formula.
