# Supplementary material for: A new metabolic signature contributes to disease progression and predicts worse survival in melanoma
Source: Bioengineered. 2020 Oct 21;11(1):1099–111. doi: 10.1080/21655979.2020.1822714 (PMC8291831; doi:10.1080/21655979.2020.1822714)
Supplement: Supplemental Material [file KBIE_A_1822714_SM0229.zip › Table S1.docx]

Table S1. 675 metabolism-related genes in the training and validation sets.

| Gene symbol | |
| --- | --- |
| UBAC1 | |
| ENTPD5 | |
| CCDC58 | |
| TMEM9B | |
| GMPPA |  |
| SLC6A9 |  |
| EGLN3 |  |
| ACOX3 |  |
| REEP6 |  |
| ACP5 |  |
| PEX13 |  |
| NQO1 |  |
| ERMAP |  |
| CITED2 |  |
| GCLM |  |
| ALDOB |  |
| GFPT1 |  |
| SERINC1 |  |
| PPOX |  |
| AP4B1 |  |
| ARHGEF12 |  |
| SDHA |  |
| SMS |  |
| GALK1 |  |
| LMO2 |  |
| HAX1 |  |
| STMN1 |  |
| ADD1 |  |
| PDHA1 |  |
| NBN |  |
| ADH1C |  |
| PDHB |  |
| PEX16 |  |
| BNIP3L |  |
| GPC4 |  |
| SSR3 |  |
| CD44 |  |
| BSG |  |
| NFS1 |  |
| PRPS1 |  |
| GCDH |  |
| REG1A |  |
| DLST |  |
| GLRX |  |
| SMOX |  |
| CHST6 |  |
| HMGCS1 |  |
| ALDH9A1 |  |
| ARTN |  |
| ECI1 |  |
| FOXJ2 |  |
| UROD |  |
| HBD |  |
| RBM5 |  |
| CA2 |  |
| PC |  |
| CYP26A1 |  |
| LDHC |  |
| BMP2K |  |
| CYP4F2 |  |
| ID2 |  |
| KAT2B |  |
| DHPS |  |
| DCXR |  |
| PSMC4 |  |
| HSD11B1 |  |
| IGSF3 |  |
| ACP1 |  |
| ATP2A2 |  |
| PTGES |  |
| PIGQ |  |
| PFKP |  |
| SNCA |  |
| G6PD |  |
| C3 |  |
| PAOX |  |
| PDK3 |  |
| SLCO1A2 |  |
| MGLL |  |
| COL5A1 |  |
| ADIPOR2 |  |
| ABCG4 |  |
| RBP1 |  |
| PEX1 |  |
| ECD |  |
| CTSB |  |
| GOT1 |  |
| PEX6 |  |
| PGD |  |
| TMBIM6 |  |
| ACAA1 |  |
| CYP2S1 |  |
| ANGPTL4 |  |
| AMACR |  |
| CLDN3 |  |
| MOSPD1 |  |
| HES6 |  |
| ACOX1 |  |
| LGALS1 |  |
| MKRN1 |  |
| LRP10 |  |
| HK2 |  |
| TTR |  |
| MTHFD1 |  |
| TPST1 |  |
| GPD1 |  |
| RDH11 |  |
| KDELR3 |  |
| RBCK1 |  |
| CA6 |  |
| AGRN |  |
| IDI1 |  |
| ABCA4 |  |
| CDK1 |  |
| SULT2B1 |  |
| VCAN |  |
| NFE2 |  |
| ALDH1A1 |  |
| HSD17B7 |  |
| ABCG2 |  |
| PECR |  |
| SLC25A38 |  |
| KEL |  |
| PYCR1 |  |
| MED24 |  |
| PEMT |  |
| ALAS2 |  |
| TAL1 |  |
| GCH1 |  |
| ANGPTL3 |  |
| KIF2A |  |
| SLC27A5 |  |
| TFDP2 |  |
| IL13RA1 |  |
| ANG |  |
| RAP1GAP |  |
| POLR3K |  |
| FBXO34 |  |
| ADIPOR1 |  |
| LCAT |  |
| SLC6A8 |  |
| PICALM |  |
| HOMER1 |  |
| RAD23A |  |
| SLC35B1 |  |
| HSDL2 |  |
| HTRA2 |  |
| RNF123 |  |
| HMBS |  |
| COPB2 |  |
| HBB |  |
| AKR1A1 |  |
| PAXIP1 |  |
| EPHX2 |  |
| PNPLA8 |  |
| TFRC |  |
| ALDH3A2 |  |
| YWHAH |  |
| CYB5A |  |
| G6PC |  |
| EPHX1 |  |
| AGL |  |
| PTGR1 |  |
| CBR3 |  |
| EXT2 |  |
| SLC2A1 |  |
| NTHL1 |  |
| FBP2 |  |
| ABCD1 |  |
| EHHADH |  |
| B3GAT3 |  |
| GAL3ST1 |  |
| GYPC |  |
| SLC22A5 |  |
| ME2 |  |
| IDH3B |  |
| PEX11G |  |
| ACSL6 |  |
| ISG20 |  |
| LCK |  |
| SAR1B |  |
| CH25H |  |
| NASP |  |
| SPAG4 |  |
| TP53INP2 |  |
| E2F2 |  |
| RRAGD |  |
| ACADM |  |
| SPTA1 |  |
| PAM |  |
| MPP1 |  |
| TALDO1 |  |
| HBZ |  |
| NCAPH2 |  |
| RIOK3 |  |
| XIST |  |
| GRHPR |  |
| DCN |  |
| SLC46A3 |  |
| BPGM |  |
| ALDH8A1 |  |
| HSD17B4 |  |
| PHYH |  |
| RXRA |  |
| EZH1 |  |
| SLC6A12 |  |
| HBQ1 |  |
| FMO1 |  |
| MIF |  |
| ELOVL5 |  |
| PHKA2 |  |
| APEX1 |  |
| SOX9 |  |
| AOC3 |  |
| GNE |  |
| ALDH3A1 |  |
| SLC30A1 |  |
| SDHC |  |
| ACADVL |  |
| KIF20A |  |
| GAPDHS |  |
| HIBCH |  |
| NCOA4 |  |
| ABCA6 |  |
| NOL3 |  |
| MDH2 |  |
| ACSS1 |  |
| MINPP1 |  |
| NPC1 |  |
| DMTN |  |
| GCLC |  |
| CYP27A1 |  |
| PRDX5 |  |
| UCP2 |  |
| ABCA8 |  |
| BBOX1 |  |
| NMT1 |  |
| TOP1 |  |
| ALDOA |  |
| GLCE |  |
| ADH5 |  |
| FAS |  |
| CLDN9 |  |
| UGP2 |  |
| RBM38 |  |
| ECI2 |  |
| GABARAPL1 |  |
| NDUFV3 |  |
| HADH |  |
| TYR |  |
| IL1R1 |  |
| ACO2 |  |
| AKR1D1 |  |
| BPHL |  |
| RAP1GDS1 |  |
| PTPRG |  |
| UGDH |  |
| ACADL |  |
| OSTC |  |
| PEX19 |  |
| SEC14L1 |  |
| HADHB |  |
| TGFBI |  |
| NSDHL |  |
| ALAD |  |
| ACSL5 |  |
| PSME1 |  |
| PEX26 |  |
| HSD3B7 |  |
| SRD5A3 |  |
| BCAT1 |  |
| DDIT4 |  |
| LPIN2 |  |
| RDH16 |  |
| SUCLG2 |  |
| CYP2J2 |  |
| PYGB |  |
| HDGF |  |
| CDA |  |
| CPT2 |  |
| ATOH8 |  |
| BMPR1B |  |
| PPP2CB |  |
| HMGCL |  |
| IDH1 |  |
| ALDH2 |  |
| ALDH7A1 |  |
| ASL |  |
| ANKZF1 |  |
| COG2 |  |
| SDC3 |  |
| HSD17B6 |  |
| TPBG |  |
| CCND3 |  |
| MET |  |
| LIPE |  |
| HSPH1 |  |
| JUP |  |
| ABCA2 |  |
| IDH2 |  |
| GLRX5 |  |
| ATP6V0A1 |  |
| ODC1 |  |
| RNF19A |  |
| SLC11A2 |  |
| STC2 |  |
| CSAD |  |
| BPNT1 |  |
| PGRMC1 |  |
| BCAR3 |  |
| HTATIP2 |  |
| SLC35A3 |  |
| ASNS |  |
| GPC1 |  |
| SOD1 |  |
| PEX7 |  |
| PROS1 |  |
| TNRC6B |  |
| CCDC28A |  |
| TXN |  |
| FBLN1 |  |
| EXT1 |  |
| FOXO3 |  |
| ACKR1 |  |
| ENO2 |  |
| OSBP2 |  |
| SAP30 |  |
| LAMP2 |  |
| OPTN |  |
| PGLS |  |
| BLVRB |  |
| PFKM |  |
| PDLIM5 |  |
| CNDP2 |  |
| DCUN1D1 |  |
| STC1 |  |
| LONP1 |  |
| SDC2 |  |
| CROT |  |
| ALDH1L1 |  |
| AURKA |  |
| ARPP19 |  |
| ACP2 |  |
| TKTL1 |  |
| G0S2 |  |
| AK3 |  |
| CACNA1H |  |
| EPHA2 |  |
| ALG1 |  |
| HNF4A |  |
| VEGFA |  |
| FMO3 |  |
| BMP6 |  |
| NNT |  |
| TTPA |  |
| CHST12 |  |
| ARL2BP |  |
| KHNYN |  |
| DIO1 |  |
| TSPAN5 |  |
| CHST2 |  |
| PGAM1 |  |
| TDO2 |  |
| GNPDA1 |  |
| SLC6A6 |  |
| SELENBP1 |  |
| SHMT2 |  |
| NR3C1 |  |
| PTGDS |  |
| SLC1A5 |  |
| EIF2AK1 |  |
| CEL |  |
| TCEA1 |  |
| MT2A |  |
| MFHAS1 |  |
| SLC7A11 |  |
| MPI |  |
| IGFBP3 |  |
| ATG4A |  |
| GSR |  |
| SUCLA2 |  |
| MOCOS |  |
| ADORA2B |  |
| ACSM3 |  |
| B4GALT7 |  |
| NR3C2 |  |
| ACAT2 |  |
| ELF3 |  |
| HS6ST2 |  |
| HSP90AA1 |  |
| PRDX6 |  |
| CLN6 |  |
| SLC23A2 |  |
| HGFAC |  |
| PMM1 |  |
| ABHD6 |  |
| PDZK1IP1 |  |
| GSTK1 |  |
| ETFDH |  |
| ABCC2 |  |
| ENPEP |  |
| CFB |  |
| MYL4 |  |
| ACSL4 |  |
| BCAR1 |  |
| ADD2 |  |
| DSC2 |  |
| GSS |  |
| HRG |  |
| MERTK |  |
| F10 |  |
| PFKFB1 |  |
| MAOA |  |
| XPO7 |  |
| FASN |  |
| PPP2R5B |  |
| BTG2 |  |
| FH |  |
| POR |  |
| SCP2 |  |
| ISCA1 |  |
| DHCR24 |  |
| EPOR |  |
| HPRT1 |  |
| P4HA1 |  |
| ENO3 |  |
| TRIM58 |  |
| CYP2E1 |  |
| GDE1 |  |
| ENO1 |  |
| HS2ST1 |  |
| VEZF1 |  |
| LHPP |  |
| B4GALT2 |  |
| ME1 |  |
| SLC12A4 |  |
| MXI1 |  |
| NINJ1 |  |
| CBR1 |  |
| SULT1B1 |  |
| DHRS7 |  |
| FUT8 |  |
| ACSM1 |  |
| GPD2 |  |
| QSOX1 |  |
| SLC25A10 |  |
| XK |  |
| TPI1 |  |
| ANK1 |  |
| IGF1 |  |
| HPGD |  |
| IGFBP4 |  |
| CHST1 |  |
| FBXO9 |  |
| DCAF11 |  |
| GALE |  |
| ABCA9 |  |
| RANBP10 |  |
| NANP |  |
| ALAS1 |  |
| HACL1 |  |
| PYGL |  |
| AKR1C3 |  |
| SLC23A1 |  |
| RBP4 |  |
| CYFIP2 |  |
| RETSAT |  |
| SDCBP |  |
| MCEE |  |
| RXRG |  |
| SLC27A2 |  |
| PIPOX |  |
| CA4 |  |
| CYP39A1 |  |
| VLDLR |  |
| ACSL1 |  |
| UBE2L6 |  |
| CD1D |  |
| GCNT2 |  |
| FAH |  |
| LHX9 |  |
| HMOX1 |  |
| CTSE |  |
| HEBP1 |  |
| CHPF2 |  |
| IDH3G |  |
| ITIH4 |  |
| GNPAT |  |
| MARK3 |  |
| ABCD3 |  |
| ERP29 |  |
| NDST3 |  |
| SERPINE1 |  |
| AHSP |  |
| TMEM176B |  |
| MBL2 |  |
| SDC1 |  |
| PPARD |  |
| SPINT2 |  |
| CHST4 |  |
| CLCN3 |  |
| LEAP2 |  |
| CDO1 |  |
| FKBP4 |  |
| BTRC |  |
| TSPO2 |  |
| HSD17B2 |  |
| GYPE |  |
| NEK7 |  |
| SLC22A4 |  |
| ACOX2 |  |
| BLVRA |  |
| PDK4 |  |
| TSTA3 |  |
| ECHS1 |  |
| UROS |  |
| HSPA5 |  |
| ARG2 |  |
| CHPF |  |
| MGST3 |  |
| CIR1 |  |
| DECR1 |  |
| TMCC2 |  |
| B3GAT1 |  |
| S100A10 |  |
| SLC22A18 |  |
| EFHC1 |  |
| AHCY |  |
| FAM162A |  |
| MCCC2 |  |
| ABCB6 |  |
| APOE |  |
| PPARA |  |
| CYP1A2 |  |
| PKM |  |
| MLYCD |  |
| SERTAD1 |  |
| NEDD4 |  |
| HCCS |  |
| CAPN5 |  |
| RCL1 |  |
| GAPVD1 |  |
| IDUA |  |
| PSMD9 |  |
| YPEL5 |  |
| LDHA |  |
| GSTZ1 |  |
| PGM2 |  |
| IRS2 |  |
| CYP46A1 |  |
| PXMP2 |  |
| DLD |  |
| MAN1A1 |  |
| NT5E |  |
| DHRS1 |  |
| DEPDC1 |  |
| METAP1 |  |
| DAAM1 |  |
| TMEM97 |  |
| AGXT |  |
| ESR1 |  |
| PLOD1 |  |
| UPB1 |  |
| HAO1 |  |
| GAD1 |  |
| TFCP2L1 |  |
| CXCR4 |  |
| RPE |  |
| HMMR |  |
| CAT |  |
| MPP2 |  |
| GART |  |
| DDAH2 |  |
| NR1H4 |  |
| GSTT2 |  |
| GSTO1 |  |
| ABCA3 |  |
| ADSL |  |
| PEX11A |  |
| FBP1 |  |
| PCBD1 |  |
| TNFRSF1A |  |
| D2HGDH |  |
| XYLT2 |  |
| PTS |  |
| PMM2 |  |
| HSD17B10 |  |
| AOX1 |  |
| TGFA |  |
| EPB42 |  |
| PAPSS2 |  |
| KLF3 |  |
| ELL2 |  |
| PINK1 |  |
| AADAT |  |
| SLC25A13 |  |
| TNS1 |  |
| ACADS |  |
| MDH1 |  |
| SUCLG1 |  |
| GOT2 |  |
| FBXO7 |  |
| HSD17B11 |  |
| ISOC1 |  |
| FECH |  |
| ECH1 |  |
| SLC37A4 |  |
| GALK2 |  |
| CENPA |  |
| HAGH |  |
| CTNS |  |
| TGFB2 |  |
| SLC25A37 |  |
| EGFR |  |
| ABCA1 |  |
| FADS2 |  |
| VNN1 |  |
| CLIC2 |  |
| GUSB |  |
| DIO2 |  |
| GLUL |  |
| SLC10A3 |  |
| GYS1 |  |
| B4GALT4 |  |
| PGK1 |  |
| FTCD |  |
| PSMB10 |  |
| CAST |  |
| IGFBP1 |  |
| CYP1A1 |  |
| INMT |  |
| CRAT |  |
| AUH |  |
| IL4I1 |  |
| CPT1A |  |
| TRAK2 |  |
| COMT |  |
| ABCA5 |  |
| CD36 |  |
| CRYZ |  |
| KYNU |  |
| EPB41 |  |
| ATXN1 |  |
| XDH |  |
| PLOD2 |  |
| TRIM10 |  |
| NFE2L1 |  |
| HDLBP |  |
| ABCD2 |  |
| FN3K |  |
| GCKR |  |
| NARF |  |
| SYNJ1 |  |
| ACAA2 |  |
| TFF3 |  |
| PTGES3 |  |
| BCKDHB |  |
| GAD2 |  |
| MBOAT2 |  |
| GPC3 |  |
| B4GALT1 |  |
| BIK |  |
| ALDH6A1 |  |
| DPYSL4 |  |
| NDRG2 |  |
| AQP9 |  |
| GMPS |  |
| IRF8 |  |
| CTH |  |
| B3GALT6 |  |
| GSTM4 |  |
| CASP6 |  |
| SLC35D1 |  |
| SDHD |  |
| ETS2 |  |
