# Supplementary material for: A new metabolic signature contributes to disease progression and predicts worse survival in melanoma
Source: Bioengineered. 2020 Oct 21;11(1):1099–111. doi: 10.1080/21655979.2020.1822714 (PMC8291831; doi:10.1080/21655979.2020.1822714)
Supplement: Supplemental Material [file KBIE_A_1822714_SM0229.zip › Table S2.docx]

Table S2. 30 metabolism-related genes with a significant survival.

| Gene symbol | |
| --- | --- |
| NQO1 | |
| LMO2 | |
| HSD11B1 |  |
| C3 |  |
| ABCA4 |  |
| PYCR1 |  |
| GCH1 |  |
| IDH3B |  |
| ISG20 |  |
| KIF20A |  |
| UCP2 |  |
| HADH |  |
| PSME1 |  |
| ABCC2 |  |
| CFB |  |
| BTG2 |  |
| HS2ST1 |  |
| CYFIP2 |  |
| CYP39A1 |  |
| UBE2L6 |  |
| SERPINE1 |  |
| TMEM176B |  |
| ABCB6 |  |
| TMEM97 |  |
| SLC25A13 |  |
| FECH |  |
| CENPA |  |
| GYS1 |  |
| IL4I1 |  |
| IRF8 |  |
