# Supplementary material for: A new metabolic signature contributes to disease progression and predicts worse survival in melanoma
Source: Bioengineered. 2020 Oct 21;11(1):1099–111. doi: 10.1080/21655979.2020.1822714 (PMC8291831; doi:10.1080/21655979.2020.1822714)
Supplement: Supplemental Material [file KBIE_A_1822714_SM0229.zip › Table S3.docx]

Table S3. Ten metabolism-related genes for constructing a model formula.

We applied used the following formula: [NQO1* 0.093] + [ABCA4*0.089] + [GCH1*(-0.164)] + [ISG20*(-0.091)] + [KIF20A*0.239] + [PSME1*(-0.269)] + [ABCC2*0.092] + [HS2ST1*(-0.23)] + [CYP39A1*(-0.069)] + [FECH*0.185].
