# Supplementary figures and images for: A new metabolic signature contributes to disease progression and predicts worse survival in melanoma
Source: Bioengineered. 2020 Oct 21;11(1):1099–111. doi: 10.1080/21655979.2020.1822714 (PMC8291831; doi:10.1080/21655979.2020.1822714)

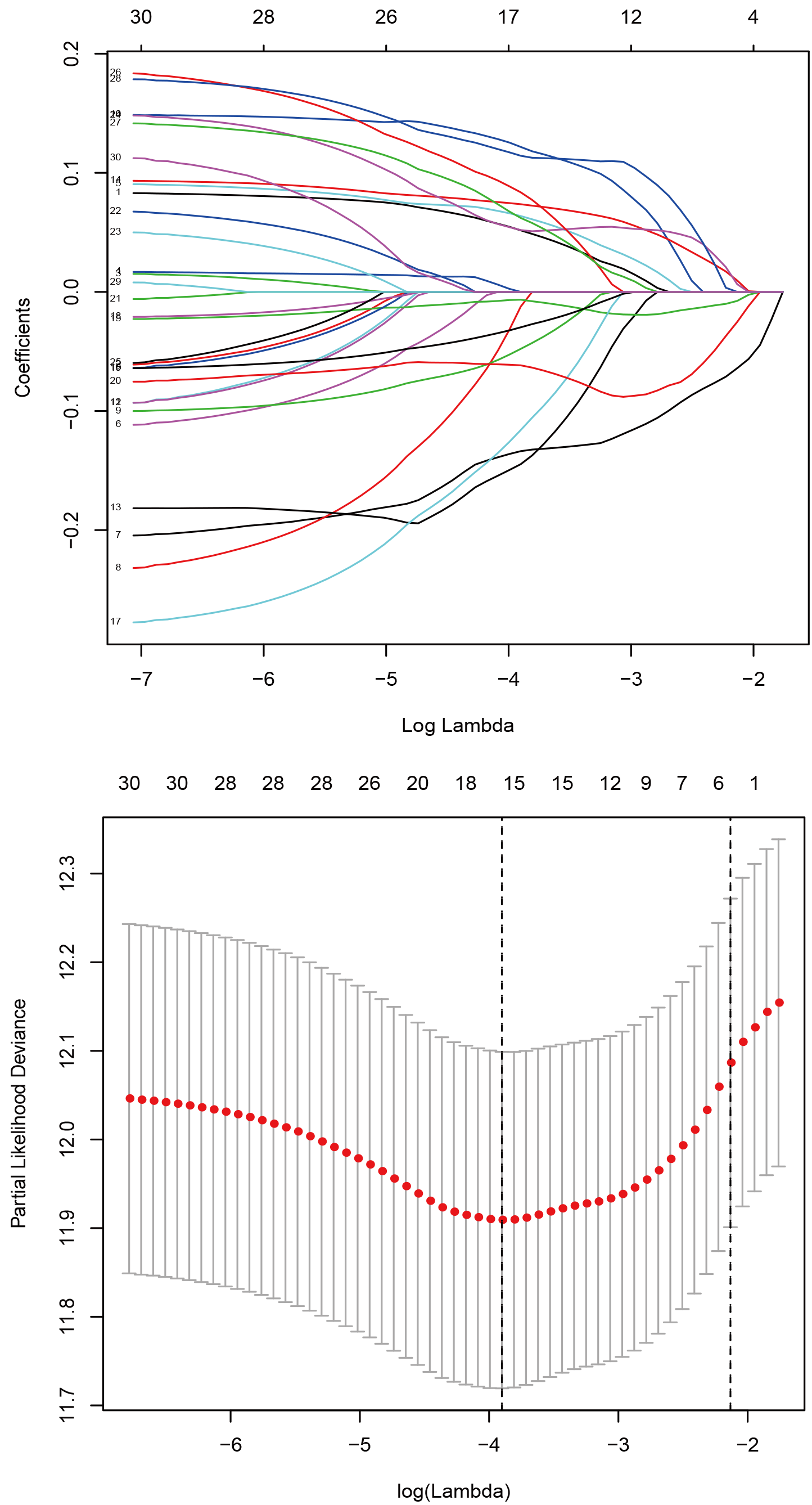

Supplement: Supplemental Material [file KBIE_A_1822714_SM0229.zip › Figure S1.tif]

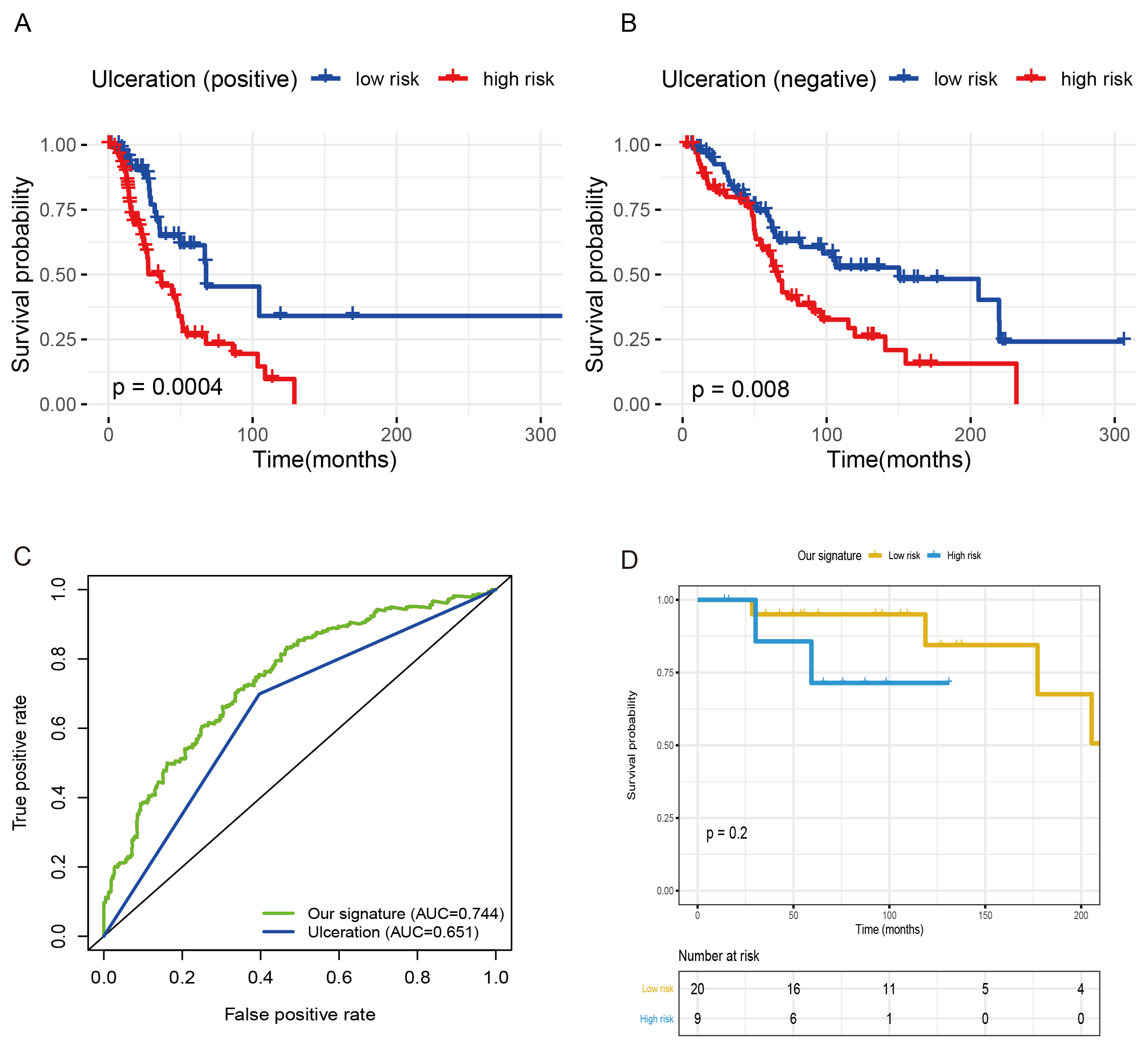

Supplement: Supplemental Material [file KBIE_A_1822714_SM0229.zip › Figure S2.tif]
